# Developmental changes in gamma-aminobutyric acid levels in attention-deficit/hyperactivity disorder

**DOI:** 10.1038/tp.2015.79

**Published:** 2015-06-23

**Authors:** S Bollmann, C Ghisleni, S-S Poil, E Martin, J Ball, D Eich-Höchli, R A E Edden, P Klaver, L Michels, D Brandeis, R L O'Gorman

**Affiliations:** 1 Center for MR-Research, University Children's Hospital Zurich, Zürich, Switzerland; 2 Neuroscience Center Zurich, University of Zurich and ETH Zurich, Zürich, Switzerland; 3 Zurich Center for Integrative Human Physiology, University of Zurich, Zürich, Switzerland; 4 Institute for Biomedical Engineering, University of Zurich and ETH Zurich, Zürich, Switzerland; 5 Centre for Advanced Imaging, University of Queensland, Brisbane, QLD, Australia; 6 Department of Child and Adolescent Psychiatry, University of Zurich, Zürich, Switzerland; 7 Psychiatric University Hospital, Zürich, Switzerland; 8 Russell H. Morgan Department of Radiology and Radiological Science, The Johns Hopkins University School of Medicine, Baltimore, MD, USA; 9 F. M. Kirby Center for Functional Brain Imaging, Kennedy Krieger Institute, Baltimore, MD, USA; 10 Department of Psychology, University of Zurich, Zürich, Switzerland; 11 Institute of Neuroradiology, University Hospital of Zurich, Zürich, Switzerland; 12 Department of Child and Adolescent Psychiatry and Psychotherapy, Central Institute of Mental Health Mannheim, Medical Faculty Mannheim/Heidelberg University, Mannheim, Germany; 13 Pediatric Research Center, University Children's Hospital Zurich, Zürich, Switzerland

## Abstract

While the neurobiological basis and developmental course of attention-deficit/hyperactivity disorder (ADHD) have not yet been fully established, an imbalance between inhibitory/excitatory neurotransmitters is thought to have an important role in the pathophysiology of ADHD. This study examined the changes in cerebral levels of GABA+, glutamate and glutamine in children and adults with ADHD using edited magnetic resonance spectroscopy. We studied 89 participants (16 children with ADHD, 19 control children, 16 adults with ADHD and 38 control adults) in a subcortical voxel (children and adults) and a frontal voxel (adults only). ADHD adults showed increased GABA+ levels relative to controls (*P*=0.048), while ADHD children showed no difference in GABA+ in the subcortical voxel (*P*>0.1), resulting in a significant age by disorder interaction (*P*=0.026). Co-varying for age in an analysis of covariance model resulted in a nonsignificant age by disorder interaction (*P*=0.06). Glutamine levels were increased in children with ADHD (*P*=0.041), but there was no significant difference in adults (*P*>0.1). Glutamate showed no difference between controls and ADHD patients but demonstrated a strong effect of age across both groups (*P*<0.001). In conclusion, patients with ADHD show altered levels of GABA+ in a subcortical voxel which change with development. Further, we found increased glutamine levels in children with ADHD, but this difference normalized in adults. These observed imbalances in neurotransmitter levels are associated with ADHD symptomatology and lend new insight in the developmental trajectory and pathophysiology of ADHD.

## Introduction

Attention-deficit/hyperactivity disorder (ADHD) is a common developmental psychiatric disorder characterized by inattention, hyperactivity and impulsivity with a prevalence of about 5%.^1^ Although traditionally considered a disorder of childhood, mounting evidence suggests that ADHD often persists into adulthood.^2^ While the neurobiological basis and developmental course of ADHD have not yet been fully established, an imbalance between inhibitory/excitatory neurotransmitters is thought to have an important role in the pathophysiology of ADHD.^3^

GABA, the main inhibitory neurotransmitter in the human cerebral cortex is synthesized from neuronal glutamate (Glu), and converted back into Glu in astrocytes. The astrocytic Glu is then catalyzed into glutamine (Gln) and transported to the presynaptic neuron, where it is cycled back into Glu, some of which is then converted into GABA.^4^ GABA has been shown to be implicated in dopaminergic neurotransmission in the striatum^5^ suggesting an important role in ADHD. Further, GABA seems to be important for behavioral inhibition and self-control,^6, 7^ as reduced GABA levels are associated with high impulsivity, less cognitive control and impaired response inhibition.

Glu, the major excitatory neurotransmitter, modulates the release of dopamine,^3^ and imbalances in Glu are believed to interfere with the gating of sensory information in striato-frontal pathways in patients with ADHD.^3^

In children with ADHD, an increase in Glu+Gln (Glx) was observed in frontal areas,^8, 9, 10^ right prefrontal cortex^10, 11^ and left striatum^3, 10, 11^ relative to controls, although these differences did not reach significance in a recent meta-analysis.^12^ Treatment with stimulant medication has been reported to decrease Glx in the basal ganglia in children with ADHD.^13^ However, there are also studies reporting no significant Glx differences with stimulant treatment.^14, 15, 16^ In ADHD adults, on one hand increases in Glx have been observed in the basal ganglia^3^ and in the left cerebellar hemisphere.^17^ On the other hand, studies report a reduction in Glx in the right anterior cingulate cortex,^18^ in a left midfrontal region^19^ and in the basal ganglia^20^ in adults with ADHD compared with controls. As the magnetic resonance spectroscopy (MRS)-visible Glx signal includes contributions from the metabolic Glx pool as well as the neurotransmitter pool, the MRS Glx signal represents only a nonspecific marker for neurotransmitter Glu. However, since neurotransmitter Glu is converted into Gln in glial cells,^21^ some authors have suggested that Gln may represent a more specific marker for neurotransmitter Glu.^22, 23, 24^

In children with ADHD, only one previous study has investigated cerebral GABA+ concentrations, reporting reduced levels in the sensorimotor cortex.^25^ However, to date, GABA+ has not been assessed in the basal ganglia in ADHD, although structural and functional basal ganglia abnormalities appear to represent a core finding in the ADHD literature, as highlighted in recent meta-analyses.^26, 27^ Further, no previous studies have investigated both inhibitory and excitatory neurotransmission in both children and adults with ADHD in a single study, and the developmental trajectories of neurotransmitter levels in ADHD remain unclear, despite the known developmental abnormalities observed in the basal ganglia.^28^

The primary aim of this study was to investigate whether inhibitory (GABA) and excitatory (Glu/Gln) neurotransmitter levels in a subcortical voxel are altered in children and adults with ADHD. A secondary aim was to investigate the relationship between neurotransmitter levels and ADHD symptomatology and how GABA, Gln and Glu change with development. For the ADHD adults, we examined a left frontal region centered on the dorsolateral prefrontal cortex in addition to the subcortical voxel. We hypothesized that GABA+ levels would be reduced in ADHD patients in the subcortical and frontal voxels, based on a recent report of decreased GABA in the sensorimotor cortex in children with ADHD.^25^ We also expected to find increased levels of Glu in children with ADHD.^8, 9, 10^

## Materials and methods

The participant group consisted of 89 participants including 16 children with ADHD and 19 control children, 16 adults with ADHD and 38 control adults. Data were acquired as part of a large developmental study investigating ADHD-related differences in GABA, Glu and Gln in the context of the normal maturational changes in these neurotransmitter systems. For this reason, a larger control group was recruited, but the groups were matched for age, handedness and gender. One ADHD adult was excluded because of recreational drug use. All participants complied with magnetic resonance imaging (MRI) safety standards. The control participants did not report any current or previous neurologic or psychiatric diagnoses or any current use of psychoactive medications. All spectra were inspected visually to assess fit quality and the signal-to-noise ratio, leading to the exclusion of one further adult ADHD patient and one control child. One child was excluded from the control group due to elevated ADHD symptom scores to avoid a confound due to subclinical cases (Table 1 and Table 2). Because of the larger adult control group, all analyses were additionally performed with a one by one matching of the control subjects to the patients based on age and gender.

The ADHD adults were recruited from the Psychiatric University Clinic Zürich and screened for comorbidities. All the patients were diagnosed according to DSM-IV criteria, by a consultant psychiatrist specialising in ADHD. To ensure a representative ADHD sample, we included participants with current mild depressive symptoms, but excluded any patients with major depression or current severe Axis I or II disorder, substance use disorder, autism spectrum disorder, tic disorder or any other medical or neurological illness affecting brain function. The ADHD children were recruited by the Department of Child and Adolescent Psychiatry of the University of Zurich. The Kiddie-Sads-Present and Lifetime clinical interview^29^ was performed with all ADHD children to ensure the diagnosis of combined ADHD and to exclude participants with comorbidities. Participants taking stimulant medications were instructed to interrupt their medication at least 72 h before the MRI measurements. Antidepressant medication in six adult ADHD participants was not interrupted. A urine toxicology test was not performed before scanning.

Symptom scores were assessed with the German version of the Conners' Adult ADHD Rating Scale^30^ and the German short form of the Wender-Utah Rating Scale.^31^ For children, we used the research version of the Conners-3D^32^ and the strengths and difficulties questionnaires in their German adaptations.^33^

After complete description of the study to the subjects, written informed consent was obtained. For the ADHD and control children, written consent was obtained from the parents and assent was obtained from the children. The study was approved by the cantonal ethics committee of Zürich and was conducted in accordance with guidelines of the Declaration of Helsinki. The participants received 60 Swiss Francs in vouchers for participation in this study.

**Figure 1:**
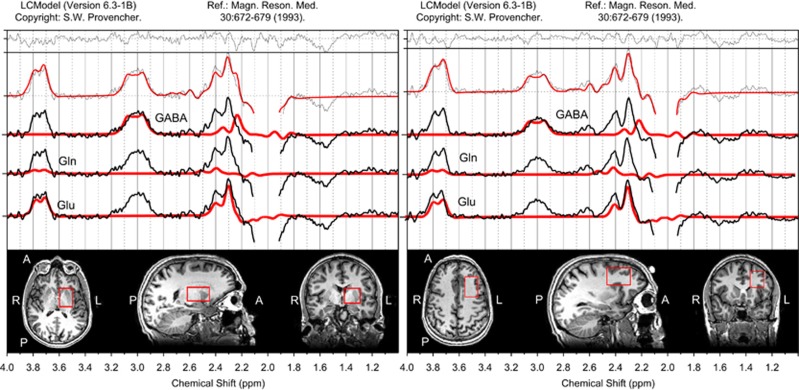
Left subcortical and left frontal voxel positions and representative spectra. The edited spectral fits and the position of the left subcortical and the left frontal voxel in a representative adult subject.

MR measurements were performed with a 3T GE HD.xt TwinSpeed MRI scanner (GE Healthcare, Milwaukee, WI, USA), using an eight-channel receive-only head coil. The MRI protocol included a T1-weighted spoiled gradient echo scan (TR=9.94 ms; TI=600 ms; FOV=256 mm × 192 mm; matrix=256 × 192; flip angle=8° axial plane; slice thickness=1 mm; 172 slices) for planning the positioning of the target voxels. GABA-edited MR spectra were acquired using the MEGA-PRESS method with TE=68 ms, TR=2000 ms, 320 averages (160 pairs) and an eight-step phase cycle. We acquired spectra from a 28 × 40 × 25 mm^3^ voxel in a subcortical region centered on the left basal ganglia (also including anterior thalamus) from all the participants. An additional 25 × 40 × 30 mm^3^ voxel was acquired in the left frontal lobe, centered on the dorsolateral prefrontal cortex from the adult participants (see Figure 1). The assessment of GABA with MEGA-PRESS is confounded by the co-editing of macromolecules which contribute to the edited peak at 3 p.p.m., so the GABA findings described subsequently represent GABA+ rather than pure GABA values.

The T1-weighted images were segmented in native space using SPM8 with the ‘New Segment' module (Wellcome Department of Cognitive Neurology, London, UK) running in MATLAB 2012b (The MathWorks, Natick, MA, USA) to correct the spectroscopy results for partial volume cerebrospinal fluid contamination and different water compartment relaxation times.^34^

The spectra were coil combined with weighting factors derived from the first point of the free induction decay signal from the unsuppressed water lines acquired with each coil. Water scaled metabolite concentrations were derived with LCModel version 6.3-1B.^35^ The edited spectra were analyzed with a simulated basis set including basis spectra for GABA, glutathione, NAA, Gln, Glu and *N*-acetyl aspartyl glutamate. GABA+/H_2_O, Glu/H_2_O and Gln/H_2_O were quantified from the edited spectra, using the control parameter sptype=‘mega-press-2'. The edited spectra provided a similar fit quality compared with the edit OFF MEGA-PRESS subspectra for Glu/H_2_O, but a better fit quality for Gln/H_2_O compared with the edit OFF subspectra (Gln detected with Cramer–Rao lower bounds (CRLB) <30% in 73/89 edited spectra and 43/89 edit OFF subspectra). The CRLB for GABA ranged from 2–10% for all participants. An estimate of general fit quality across each spectrum was assessed from the CRLB of the NAA fit, both in the edited spectra and in the edit OFF subspectra (edited spectra: NAA CRLB=1–3%, edit OFF subspectra: NAA CRLB=1–5% for all cortical and subcortical spectra).

Statistical analysis was performed using R 3.1.1 (ref. 36) and ggPlot2.^37^ Normality of the data was assessed using Quantile–Quantile plots of the analysis of variance (ANOVA) residuals. ADHD-related and developmental differences in subcortical GABA+ and Glu were assessed with a 2 × 2 ANOVA, including age group (adults and children) and diagnostic group (ADHD and control) as fixed factors. *Post hoc t*-tests were performed to clarify the group differences. As the Gln levels were not normally distributed, differences between ADHD and control participants were tested using a two-tailed Wilcoxon rank-sum test. Correction for multiple comparisons was performed with Benjamini and Hochberg correction, with a false discovery rate (FDR) of 0.1.^38^ A multiple regression model was used to investigate the relationship between metabolite levels and ADHD symptom scores from the Conners' inventory, age, methylphenidate dose and gender, using the normally distributed data. To construct the final model, we started with the full model including gender, age, dose, inattention/memory-score, hyperactivity-score, impulsivity-score and the self-concept-score, then removed correlated regressors and finally removed nonsignificant predictors in a stepwise manner until only significant predictors remained in the model.

## Results

### GABA+/H_2_O—subcortical

The gray matter content of the subcortical voxel did not differ significantly between controls (*M*=52%, s.d.=3%) and ADHD patients (*M*=50%, s.d.=5% *P*>0.1). However, there was a significant difference in gray matter content comparing adults (*M*=51%, s.d.=4%) to children (*M*=55%, s.d.=4% *P*<0.001).

**Figure 2:**
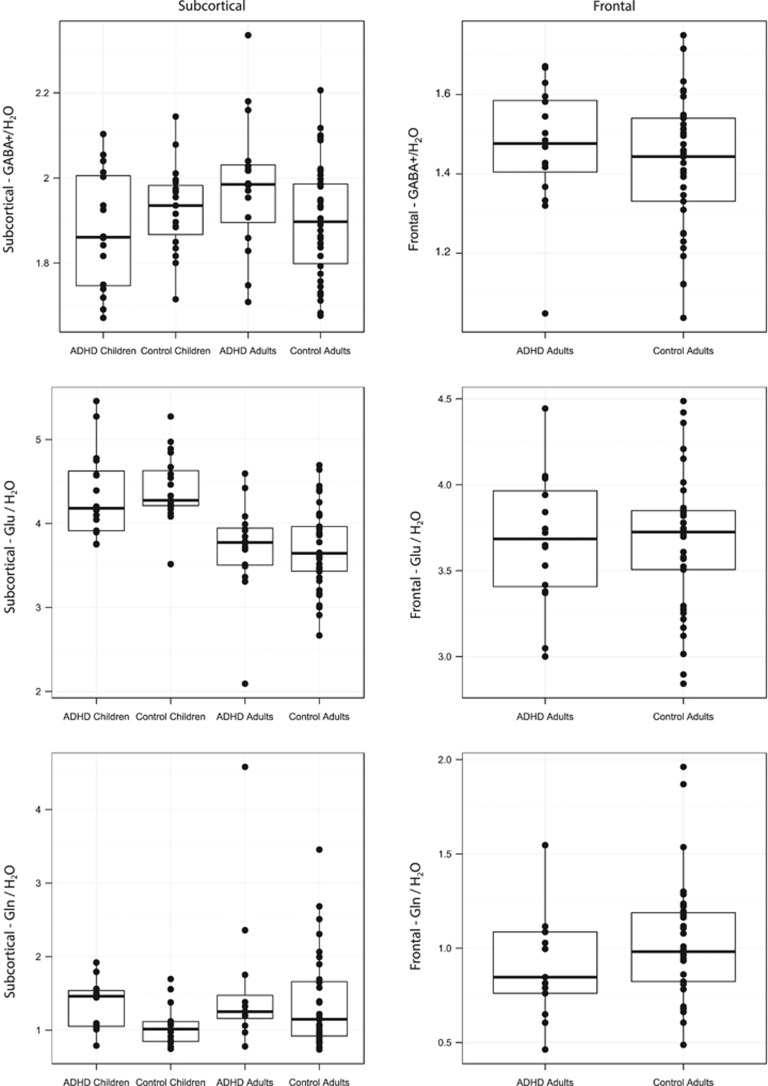
GABA+, Glu and Gln levels in the left subcortical and in the left frontal voxel GABA+/H_2_O (top row), Glu/H_2_O (middle row) and Gln/H_2_O (bottom row) ratios from ADHD children, control children, ADHD adults and control adults in the subcortical voxel (left column) and in the left frontal voxel (right column). ADHD, attention-deficit/hyperactivity disorder; Gln, glutamine; Glu, glutamate.

Comparing GABA+/H_2_O levels in the subcortical voxel in children and adults with and without ADHD revealed a significant age group by diagnostic group interaction (F(1,85)=5.11, *P*=0.026, *ω*^2^=0.04; Figure 2), which remained significant after FDR correction for multiple comparisons. *Post hoc t*-tests demonstrated that ADHD adults had higher GABA+/H_2_O values (*M*=1.98, s.d.=0.20) compared with controls (*M*=1.87, s.d.=0.17). This difference was significant, *t*(52)=2.03, *P*=0.048, *d*=0.56. ADHD children showed no significant differences in GABA+/H_2_O values in the subcortical voxel (*M*=1.85, s.d.=0.17) compared with controls (*M*=1.91, s.d.=0.13), *t*(33)=−1.25, *P*>0.1, *d*=0.43.

The same analysis was repeated with the one by one matched adult control group, resulting in similar results for the interaction (F(1,63)=5.56, *P*=0.021, *ω*^2^=0.06), but the comparison between GABA levels in ADHD adults and controls revealed only a trend level difference (*t*(30)=1.99, *P*=0.055, *d*=0.73), although the effect size was comparable to that observed in the full control group. Co-varying for age in an analysis of covariance model resulted in a nonsignificant age group by diagnostic group interaction in the full group (F(1,84)=3.5, *P*=0.06) but was significant in the one by one matched group (F(1,62)=5.4, *P*=0.023).

The multiple regression in adults yielded standardized betas of 0.33 for age (*P*=0.017) and 0.35 (*P*=0.010) for self-concept with an adjusted *R*^2^ of 0.26.

The regression analysis in children did not show any significant effects. There were no significant differences in estimated fit error (Cramer–Rao lower bounds) between the groups.

### Glu/H_2_O—subcortical

Comparing Glu/H_2_O differences in the subcortical voxel in children and adults in a 2 × 2 ANOVA revealed a significant main effect of age group, F(1,85)=40.6, *P*<0.001, *ω*^2^=0.31. The *post hoc t*-test revealed that children have significantly increased Glu/H_2_O values, *t*(78)=−6.59, *P*<0.001, *d*=1.49 (see Figure 2) relative to adults. The main effect of age on Glu/H_2_O levels remained significant after FDR correction for multiple comparisons. No significant difference between controls and ADHD in Glu/H_2_O values in the subcortical voxel was observed, either for adults (*t*(52)=0.07, *P*>0.1, *d*=0.02) or for children (*t*(33)=−0.45, *P*>0.1, *d*=0.16).

The Glu/H_2_O regression model in adults and children yielded no significant effect. There were no significant differences in fit errors between ADHD and controls for Glu. However, Glu fit errors were significantly higher in adults (Mdn=5.4%) compared with children (Mdn=3.8%), *W*=1477, *P*<0.001, *r*=0.49.

The same analysis was repeated with the one by one matched adult control group, resulting in similar results for the age group effect (F(1,63)=33.25, *P*<0.001, *ω*^2^=0.33). The comparison between adults and children was still significant *t*(64)=−5.87, *P*<0.001, *d*=−1.47.

### Gln/H_2_O—subcortical

The CRLB for Gln were <30% in 15/19 control children (range 13–23%), 14/16 ADHD children (range 10–22%), 32/38 control adults (range 10–29%) and 12/16 ADHD adults (range 11–28%). Using a stricter cutoff of 20% would result in the exclusion of two additional control children, two ADHD children, 15 control adults and six ADHD adults.

ADHD adults (Mdn=1.25) showed no significant difference in Gln/H_2_O compared with control adults (Mdn=1.15), *W*=229.0, *P*>0.1, *r*=0.14. However, ADHD children (Mdn=1.46) showed a significantly higher Gln/H_2_O level than control children (Mdn=1.01), *W*=152.0, *P*=0.041, *r*=0.38, which remained significant after FDR correction for multiple comparisons. There was no significant difference in Gln levels between adults (Mdn=1.2) and children (Mdn=1.09), *W*=726.0, *P*>0.1, *r*=0.11.

The Gln/H_2_O regression model was not performed due to non-normality of the data. There were no significant differences in fit errors between ADHD and controls for Gln. However, Gln fit errors were significantly higher in adults (Mdn=19.5%) compared with children (Mdn=16.00%) *W*=918.0, *P*=0.006, *r*=0.32.

The same analysis was repeated with the one by one matched adult control group, where ADHD adults (Mdn=1.25) showed no significant difference in Gln/H_2_O compared with control adults (Mdn=1.22), *W*=84.0, *P*>0.1, *r*=0.06. There was no significant difference in Gln levels between adults (Mdn=1.25) and children (Mdn=1.09), *W*=449.0, *P*>0.1, *r*=0.2.

### GABA+/H_2_O—frontal

The gray matter content of the frontal voxel did not differ significantly between controls (*M*=34%, s.d.=3%) and ADHD patients (*M*=33%, s.d.=4% *P*>0.1).

In the left frontal voxel, ADHD adults did not differ in GABA+/H_2_O values (*M*=1.47, s.d.=0.16) compared with controls (*M*=1.42, s.d.=0.17), *t*(51)=0.95, *P*>0.1, *d*=0.26 (see Figure 2). This was also the case for the one by one matched group (*t*(30)=0.02, *P*>0.1; *d*=0.01).

The multiple regression in adults yielded standardized betas of 0.58 for inattention/memory (*P*=0.003) and −0.37 (*P*=0.05) for hyperactivity with an adjusted *R*^2^ of 0.15. There were no significant differences in GABA+ fit errors between ADHD and controls (*P*>0.1).

### Glu/H_2_O—frontal

ADHD adults showed no difference in Glu/H_2_O values in the frontal voxel (*M*=3.7, s.d.=0.4) compared with controls (*M*=3.7, s.d.=0.4), *t*(51)=0.12, *P*>0.1, *d*=0.03. This was also the case for the one by one matched group (*t*(30)=−1.07, *P*>0.1; *d*=0.39).

The multiple regression analysis in adults yielded no significant effects. There were no significant differences in Glu fit errors between ADHD and controls (*P*>0.1).

### Gln/H_2_O—frontal

For this analysis, we excluded in total five Gln values with an estimated fit error (Cramer–Rao lower bound) >30% (three ADHD adults, two control adults) leaving in total 48 adults (13 patients) in the analysis.

ADHD adults showed no difference in Gln/H_*2*_O values in the frontal voxel (Mdn=0.85) compared with controls (Mdn=0.98), *W*=179.0, *P*>0.1, *r*=0.16. This was also the case for the one by one matched group (*W*=65, *P*>0.1; *d*=0.24).

The multiple regression analysis in adults was not performed due to non-normality of the data. There were no significant differences in Gln fit errors between ADHD and controls (*P*>0.1).

## Discussion

The present study examined GABA+, Glu and Gln concentrations in a subcortical voxel centered on the basal ganglia for the first time in a developmental group of children and adults with and without ADHD, demonstrating a developmental alteration in GABA+ levels in ADHD patients. GABA+ levels in the subcortical voxel were significantly correlated with ADHD symptom scores in adults. In addition, Gln levels were significantly increased in ADHD children but not in adults.

The significant interaction between age group and disorder group in GABA+/H_2_O in the subcortical voxel was driven by increased GABA+/H_2_O concentrations in ADHD adults compared with control adults, in combination with mildly reduced GABA+/H_2_O concentrations in ADHD children compared with control children. Although the lower GABA+ levels in ADHD children are broadly consistent with reports in the existing ADHD MRS literature,^25^ the increased GABA+ concentration in ADHD adults was unexpected. The GABA+ regression analysis revealed positive regression coefficients in adults for age and self-concept in the subcortical voxel. In the frontal voxel, GABA+ was positively correlated with inattention/memory but negatively correlated with hyperactivity in adults.

Taken together with the observed group differences in GABA+ and the significant age by disorder interaction, these results provide evidence for a developmental alteration in subcortical GABA+ levels in ADHD patients. The neurobiological basis for this developmental change in GABA+ levels is unknown, but may result from the progression of ADHD with brain maturation. The developmental change in subcortical GABA+ levels is also broadly consistent with results from structural MRI studies, which on one hand report decreases in gray matter in the caudate and other basal ganglia structures in children which show an apparent normalization with development and treatment;^26, 28, 39, 40, 41^ whereas on the other hand, subtle decreases in caudate gray matter in adults with ADHD compared with controls can still be observed.^42^ In the present study, the subcortical voxel did not show any significant differences in gray matter content between the ADHD and control groups, indicating that the apparent differences in GABA+ observed in this region are independent of structural differences which may arise from developmental or treatment effects. Therefore, the observed developmental changes in GABA+ seem to relate to functional rather than structural differences. Further, the regression results suggest that developmental changes in GABA+ levels may at least partly underlie the apparent change in ADHD symptomatology from childhood to adulthood.

It is possible that the significant difference in GABA between ADHD adults and controls might be driven by age differences. When age was included as a covariate in the GABA+ ANOVA in adults, the age by disorder interaction altered from F(1,85)=5.11, *P*=0.026 to F(1,84)=3.5, *P*=0.06. In the one by one matched group, the interaction was significant in the ANOVA (F(1,63)=5.56, *P*=0.021) and the analysis of covariance (F(1,62)=5.43, *P*=0.023). However, the effect of age on GABA+ is not yet clear, as some studies report a decline of GABA+ with age,^43^ while others observed an increase of GABA+ with age.^44^ The reported increase of GABA+ has also been reported to be driven by an increase of the co-edited macromolecules with age and not GABA.^44^

We did not find increased subcortical Glu levels in ADHD patients compared with controls, as found in some previous studies^10, 11, 13, 45^ but not in others.^12, 25, 46^ However, most previous studies investigated the ratio of Glx (the sum of Glu+Gln) to creatine,^3, 11, 15, 16, 47^ rather than concentrations of Glx or Glu. Ratios to creatine are simpler to derive since they do not require correction for partial volume effects arising from cerebrospinal fluid in the MRS voxel, but the assessment of Glx/Cr ratios may be confounded by alterations in striatal Cr levels, which have been reported both in children^10^ and adults^20^ with ADHD. To our knowledge, only two previous studies investigated Glx or Glu in the left basal ganglia or striatum in ADHD, reporting an increase of Glx and Glu in children aged 8 years,^10^ and a decrease in Glx in both medication naive and medicated adults.^20^ These two findings point towards a possible age interaction with Glu in the striatum, such that Glu may be increased in children with ADHD and decreased with age, resulting in lower Glu levels in adult ADHD patients. However, in the present study, we did not observe a significant interaction between subcortical Glu levels and age or a significant effect in the regression analysis, although we did observe a significant main effect of age for Glu/H_2_O in the subcortical voxel, with children showing higher Glu concentrations than adults. Our regression analysis also suggests that Glu/H_*2*_O decreases with age in children and adults, consistent with the age decrease of Glx/Cr reported by Gao *et al.,*^43^ although their results were derived from frontal and parietal regions.

To put our results into context with those from the literature, we identified 10 previous studies investigating Glx or Glx/Cr in the left basal ganglia/striatum in ADHD children or adults.^3, 10, 11, 13, 15, 16, 20, 45, 47, 48^ Two of these studies did not include a healthy comparison group^13, 45^ and one did not report group means or effect sizes for Glx,^48^ but for the remaining seven studies, we calculated the effect size (Cohen's D) from the difference between group means (ADHD–control) and the pooled standard deviations for Glx/Cr or Glx. Where data were available for multiple cohorts (for example, medicated vs unmedicated or ADHD combined vs inattentive subtype), the effect sizes were calculated separately for each ADHD cohort relative to the control group. Effect size data are shown graphically in Supplementary Figure 1. For the Glx/Cr ratios, studies showed either small positive or negative effect sizes (both in children and adults), although two studies showed a large increase in Glx/Cr in ADHD children or young adults relative to a control group.^3, 11^ For the Glx concentration, data are only available for a small number of studies, but children with ADHD tended to show positive effect sizes (increased Glx) while adults with ADHD show smaller positive or negative effect sizes (reduced Glx^20^), but further studies would be needed to corroborate and extend this finding.

To our knowledge, the present study is the first to examine Gln changes in a developmental cohort of adults and children with ADHD. We found increased Gln/H_2_O in the subcortical voxel in children with ADHD, but there were no significant differences in Gln/H_2_O between ADHD and control adults. As Gln contributes to the Glx signal, the increased Gln levels are consistent with results from a previous study reporting increased Glx levels in children with ADHD^10, 11^ (Supplementary Figure 1). Assuming that Gln represents a marker for neuronal Glu, the developmental trajectory of Gln levels observed here points towards an increase in excitatory neurotransmission in children with ADHD, which normalizes with brain maturation. While the underlying basis of this apparent increase in Gln remains unclear, the production and subsequent pruning of excitatory synapses during late childhood and adolescence could result in transiently increased Gln either from a developmental lag or from a compensatory effect in response to synaptic pruning. However, developmental changes in neurotransmitter activity are likely to result from a complex interplay between genetic and environmental factors including activity-dependent increases in synaptic strength and neuroplastic changes during adolescence. Further studies would be needed to elucidate the basis for this apparent change in Gln.

In the frontal voxel, there was no significant difference between ADHD and control adults in GABA+/H_2_O, Glu/H_2_O or Gln/H_2_O.

Several methodological limitations are inherent in the detection of GABA with MEGA-PRESS MRS. Due to the low cerebral concentration of GABA, a large MRS voxel is required for sufficient sensitivity and signal-to-noise ratio, which limits the regional specificity of the measurement. In addition, the assessment of GABA with MEGA-PRESS is confounded by the co-editing of macromolecules which contribute to the edited peak at 3 p.p.m. Therefore, we cannot exclude the possibility that the differences in GABA observed in the present study are confounded by differences in the co-edited macromolecules between ADHD patients and controls, but future studies using a MEGA-PRESS protocol with reduced macromolecular contamination^49^ may be able to clarify further the spectroscopic differences in GABA in ADHD.

Although the developmental effects observed in subcortical GABA+ levels and the significant difference in Gln levels in children with ADHD relative to control children survived correction for multiple comparisons with a relatively lenient FDR threshold of 0.1, these results did not survive correction with a more stringent FDR threshold of 0.05. Given the sample size, the relatively modest significance levels are surprising, but may result from the large variance seen both within and across age and diagnostic groups (Figure 2). Alternatively, the modest effect size may also be influenced by variability within diagnostic groups, as evidenced by the relatively wide range of symptom scores particularly in the adult ADHD group, which included some patients with lower symptom scores indicative of a milder phenotype. Although the groups were matched for gender, the inclusion of female participants may also contribute to the large group variance, as GABA+ and Glu have been shown to vary significantly with hormonal effects and throughout the menstrual cycle.^50^ However, in our sample, the variance of males and females did not differ for GABA+ in ADHD (F(1,7)=4.7, *P*=0.06) or control adults (F(1,18)=1.5, *P*=0.39). The handedness was also carefully balanced between groups but the inclusion of both left- and right-handed participants may also contribute to the group variance. Future multicenter or meta-analytic studies with larger group sizes, or groups of male, right-handed participants only may be able to elucidate further the developmental and ADHD effects observed in the present study.

In addition, medication effects could introduce variance, even though participants taking stimulants were asked to withdraw from treatment for 72 h before the MRS session. While some studies have reported a decrease in Glx levels following stimulant treatment,^45^ others showed no change in Glx between medicated and medication naive adults with ADHD.^20^ However, in addition to psychostimulant medication for ADHD, four adult participants also reported current or previous use of antidepressants within 1 month of the MRS measurement, for sleep problems. As antidepressants have been shown to alter Glx^51^ and GABA levels,^52^ to assess the potentially confounding effects of antidepressant treatment on the MRS results, the statistical comparisons were repeated after excluding all participants on antidepressants. In this smaller subsample, the developmental GABA+/H_2_O interaction was of borderline significance with a similar effect size (F(1,80)=3.93, *P*=0.05, *ω*^2^=0.03) compared with F(1,85)=5.11, *P*=0.026, *ω*^2^=0.04 in the full group. The differences in GABA+/H_2_O between ADHD and control adults were not significant anymore due to the smaller sample size, but showed a similar effect size (*t*(47)=1.58, *P*=0.1, *d*=0.46 compared with *t*(52)=2.03, *P*=0.048, *d*=0.56). Therefore, while the results do not appear to be driven by medication effects, the variability in medication status of the ADHD adults does represent a significant source of variance, and these results should be reproduced in a sample of medication naive patients.

LCModel offers improved reproducibility for GABA detection relative to alternative spectral analysis methods,^53, 54^ but with the default settings the baseline fit can include a large fraction of the GABA peak, introducing an error into the estimated GABA concentrations. However, with the MEGA-PRESS-2 and MEGA-PRESS-3 control parameters (available from version 6.3 onwards), the default baseline fitting in LCModel is turned off and this error is reduced.

Although the CRLB cutoff used for Gln was relatively lenient (30%), using a stricter cutoff of 20% resulted in the exclusion of four additional children (two ADHD children and two control children). In this smaller subset of participants, the group difference in Gln in children was no longer significant (*W*=112.0, *P*=0.137, *r*=0.30, compared with *W*=152.0, *P*=0.041, *r*=0.38 in the full group). However, as higher CRLB are expected for lower concentration metabolites, using a strict CRLB cutoff for low-concentration metabolites like Gln and GABA can introduce a systematic bias toward higher concentrations as lower concentrations are more likely to be excluded. This potential bias can confound the analysis of group differences and should be considered carefully when selecting a CRLB cutoff.

## Conclusion

ADHD patients show altered levels of GABA+ in the subcortical voxel (centered on the basal ganglia) which change with development. Children with ADHD also show increased Gln levels in the subcortical voxel, but this difference normalized in adults. These imbalances in neurotransmitter levels are associated with symptom scores and lend new insight in the development and pathophysiology of ADHD.

## Figures and Tables

**Table 1:** Demographic and clinical characteristics of the adult participants

	n	*Mean (s.d.)*	*Median*	*Range*	P
*Age*					
ADHD (m/f)	8/8	38.4 (11.8)	37.6	23.7–61.1	0.04^a^
Control (m/f)	19/19	31.6 (9.2)	27.5	21.0–50.6	

*WURS-k Sum*					
ADHD	16	33.4 (11.1)	34	13.0–51.0	<0.001^a^
Control	30	12.9 (10.4)	10	0.0–38.0	
					
*CAARS-S:L (ADHD-Index)*					
ADHD	16	63.0 (8.4)	62	51.0–79.0	<0.001^a^
Control	30	44.9 (5.7)	43	36.0–62.0	

*CAARS-O:L (ADHD-Index)*					
ADHD	13	64.6 (11.8)	63	49.0–83.0	<0.001^b^
Control	28	46.6 (6.1)	48	37.0–58.0	

*Medication*					
ADHD	Methylphenidate (*n*=14); trazodone (*n*=2)^c^; dexmethylphenidate (*n*=2); bupropion (*n*=1); agomelatine (*n*=1); amitriptyline (*n*=1)^c^; prednisone (*n*=1); oral contraceptives (*n*=3)				
Control	Finasteride (*n*=1); fluorouracil (*n*=1)^d^; salicylic acid (*n*=1); paracetamol (*n*=1); minoxidil (*n*=1); oral contraceptives (*n*=8)				

*Comorbidities*					
ADHD	Sleep problems (*n*=1)				
Control	None				

*Handedness*					
ADHD (r/l)	13/3				
Control (r/l)	32/6				

Abbreviations: ADHD, attention-deficit/hyperactivity disorder; CAARS, Conners' Adult ADHD Rating Scales; f, female; l, left-handed; m, male; n, number of participants; r, right-handed; WURS, Wender Utah Rating Scale.

a Two-tailed Wilcoxon rank-sum test.

b Two-tailed two-sample *t*-test.

c Current or previous reported use for sleep problems.

d Prescribed for skin warts.

**Table 2:** Demographic and clinical characteristics of the children participants

	n	*Mean (s.d*.)	*Median*	*Range*	P
*Age*					
ADHD (m/f)	9/7	10.8 (1.1)	10.7	8.7–13.1	0.89^a^
Control (m/f)	11/8	10.8 (1.9)	11	8.1–14.7	
					
*Conners Parents ADHD-Index*					
ADHD	16	69.8 (3.1)	70	61.0–73.0	<0.001^b^
Control	19	46.9 (12.6)	46	20.0–63.0	
					
*SDQ Parents Total*					
ADHD	15	19.1 (6.5)	18	10.0–34.0	<0.001^a^
Control	19	5.1 (3.1)	5	1.0–14.0	
					
*Medication*					
ADHD	Methylphenidate (*n*=11)				
Control	None				
					
*Comorbidities*					
ADHD	F40.2 specific (isolated) phobias (*n*=4); F82 specific developmental disorder of motor function (*n*=2); F81.0 specific reading disorder (*n=*1); F81.2 specific disorder of arithmetical skills (*n*=1)				
Control	None				
					
*Handedness*					
ADHD (r/l)	12/4				
Control (r/l)	18/1				

Abbreviations: ADHD, attention-deficit/hyperactivity disorder; f, female; l, left-handed; m, male; n, total number of participants; r, right-handed; SDQ, strengths and difficulties questionnaire.

a Two-tailed two-sample *t*-test.

b Two-tailed Wilcoxon rank-sum test.
