# Supplementary figures and images for: Developmental changes in gamma-aminobutyric acid levels in attention-deficit/hyperactivity disorder
Source: Transl Psychiatry. 2015 Jun 23;5(6):e589–. doi: 10.1038/tp.2015.79 (PMC4490289; doi:10.1038/tp.2015.79)

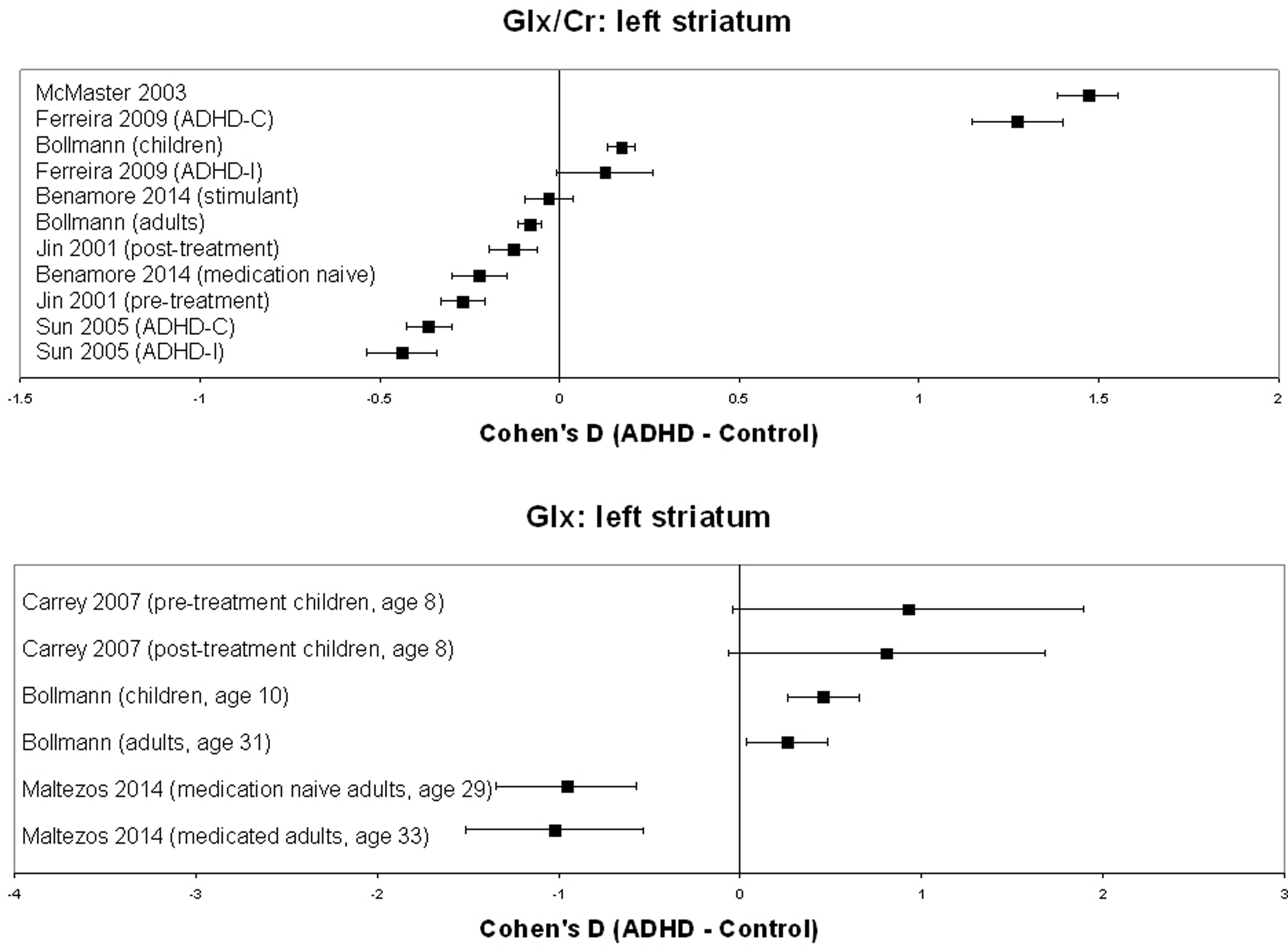

Supplement: Supplementary Figure 1 [file tp201579x1.tif]
