# Supplementary material for: Developmental changes in gamma-aminobutyric acid levels in attention-deficit/hyperactivity disorder
Source: Transl Psychiatry. 2015 Jun 23;5(6):e589–. doi: 10.1038/tp.2015.79 (PMC4490289; doi:10.1038/tp.2015.79)
Supplement: Supplementary Figure 1 Legend [file tp201579x2.doc]

Supplementary figure 1:

Forest plot showing the effect size (Cohen’s D) from the difference in group means (ADHD > Control) for the Glx/Cr ratios (top) and the Glx concentrations (bottom). Where multiple ADHD cohorts were examined the effect size is calculated separately for each cohort (ADHD-C: ADHD combined type; ADHD-I: ADHD-inattentive type). The 95% confidence intervals (CI) for each effect size are depicted with horizontal lines.
